# Natural products targeting the MAPK-signaling pathway in cancer: overview

**DOI:** 10.1007/s00432-023-05572-7

**Published:** 2024-01-09

**Authors:** Aiwen Shi, Li Liu, Shuang Li, Bin Qi

**Affiliations:** Changchun University of Chinese Medicine, School of Phharmacy, 1035 Boshuo Road, Jingyue Street, Nanguan District, Changchun City, Jilin Province China

**Keywords:** MAPK, Natural compounds, Cancer, Phytochemicals

## Abstract

**Purpose:**

This article summarizes natural products that target the MAPK-signaling pathway in cancer therapy. The classification, chemical structures, and anti-cancer mechanisms of these natural products are elucidated, and comprehensive information is provided on their potential use in cancer therapy.

**Methods:**

Using the PubMed database, we searched for keywords, including “tumor”, “cancer”, “natural product”, “phytochemistry”, “plant chemical components”, and “MAPK-signaling pathway”. We also screened for compounds with well-defined structures that targeting the MAPK-signaling pathway and have anti-cancer effects. We used Kingdraw software and Adobe Photoshop software to draw the chemical compound structural diagrams.

**Results:**

A total of 131 papers were searched, from which 85 compounds with well-defined structures were selected. These compounds have clear mechanisms for targeting cancer treatment and are mainly related to the MAPK-signaling pathway. Examples include eupatilin, carvacrol, oridonin, sophoridine, diosgenin, and juglone. These chemical components are classified as flavonoids, phenols, terpenoids, alkaloids, steroidal saponins, and quinones.

**Conclusions:**

Certain MAPK pathway inhibitors have been used for clinical treatment. However, the clinical feedback has not been promising because of genomic instability, drug resistance, and side effects. Natural products have few side effects, good medicinal efficacy, a wide range of sources, individual heterogeneity of biological activity, and are capable of treating disease from multiple targets. These characteristics make natural products promising drugs for cancer treatment.

## Introduction

Cancer is a serious health challenge globally, with 2020 statistics showing that more than 19 million new cases and approximately 10 million deaths occurred. It is estimated that new cancer cases worldwide will increase by approximately 50% in 20 years (Mao et al. 2022). Currently, breast cancer is the most common cancer, with approximately 2.3 million new patients each year. The next most frequent types are lung, colorectal, prostate, and stomach cancers. However, lung cancer is the leading cause of cancer-related mortality, with approximately 1.8 million deaths each year, followed by colorectal, liver, stomach, and breast cancers (Sung et al. 2021). Evidence from clinical research suggests that a poor diet, obesity, and insufficient exercise habits may increase cancer risk. Many cancer cases can be treated, with current common treatment methods, including chemotherapy, hormone therapy, immunotherapy, and targeted therapy (Siegel et al. 2022; Rock et al. 2022). Therefore, identifying new therapeutic agents with specific effects on different tumor types is necessary.

MAPK pathway is a highly conserved tertiary kinase model (He and Meng 2020) that is mainly composed of MAPKKK, MAPKK, and MAPK (Park and Baek 2022). Intracellular and extracellular signals stimulate the upstream kinase MAPKKK, which responds by activating the intermediate kinase MAPKK. This then activates the downstream kinase MAPK (Lee et al. 2020). In mammals, there are more than a dozen proteins in the model of the tertiary kinase of MAPK. The four most common subprotein families include the ERK1/2, JNK, p38, and ERK5 families (Cargnello and Roux 2011). MAPK pathway signaling can impact many biological processes in eukaryotic cells and can regulate different cellular activities, including proliferation, differentiation, and migration, by transducing extracellular signals (Guo et al. 2022; Kent et al. 2020; Brägelmann et al. 2021).

However, to our knowledge, no preclinical or clinical research on natural products targeting MAPK-signaling pathways in cancer has been published. Therefore, this article summarizes the components of natural products and the role of the MAPK pathway in various cancers, as well as elucidating the mechanisms of their potential use for cancer treatment.

## Major MAPK-signaling pathways

The three main MAPK-related-signaling pathways in cells are the classical MAPK pathway, the JNK and p38 MAPK pathway, and the ERK5 pathway.

Overactivation of the classical MAPK pathway (Ras/RAF/MEK/ERK (MAPK) pathway) leads to more than 40% of cancer cases (Yuan et al. 2020). RAS is a gene family that is commonly mutated in cancer. The most frequent mutation is KRAS, such as pancreatic ductal adenocarcinomas and colorectal carcinomas (Drosten and Barbacid 2020). RAS is usually activated on the membrane downstream of the growth factor receptors. RAS contains three gene isoforms: H-RAS, K-RAS, and N-RAS. Although they have highly homologous sequences, they have distinct functions that lead to different physiological functions (Moore et al. 2020; Yuan et al. 2020). RAF protein family kinases include three isoforms: RafA, RafB, RafC, as well as two close pseudokinases (KSR1/2). In addition, BRAF mutations occur in approximately 8% of cancers, which are very common in melanomas (Drosten and Barbacid 2020). In this classical pathway, the RAS mutation rate is the highest (22%), followed by BRAF (8%) and MEK (< 1%), while ERK mutations are sporadic (Yaeger and Corcoran 2019). The three-layered MAPK-signaling cascade is initiated when RTK and RAS are activated. The three RAF subtypes and downstream MEK1/2 and ERK1/2 from a constitute-signaling module to guide a series of physiological functions (Ullah et al. 2022).

The p38 MAPK pathway plays essential roles in signaling cascade responses. In mammals, it has four p38 kinase members: p38α, p38β, p38γ, and p38δ. The expression patterns of the upstream activator and downstream effector differ (Cheng et al. 2020). p38 MAPK is involved in regulating cell proliferation, growth, and apoptosis. It is usually activated by MKK3 and MKK6 kinases, but can also be phosphorylated through MKK4 kinase, which is an activator of JNK. When the p38 protein is activated, it is usually transferred from the cytoplasm to the nucleus to regulate downstream-signaling molecules (Sui et al. 2014). JNK has three subtypes: JNK1, JNK2, and JNK3. JNK1 and JNK2 are widely distributed in tissues, but JNK3 is mainly limited to neuronal tissues, testis, and cardiac myocytes (Cargnello and Roux 2011). They respond to various stressors, such as DNA-damaging agents and oxidative stress (Xie et al. 2018). Activation of JNK MAPK is similar to the p38 MAPK process, mainly occurring via upstream kinases MKK4 and MKK7. This requires dual phosphorylation of Thr and Tyr residues within a conserved Thr–Pro–Tyr motif in their activation loops (Cargnello and Roux 2011). This supports the regulation of various cellular processes, including autophagy, differentiation, and proliferation.

**Fig. 1:**
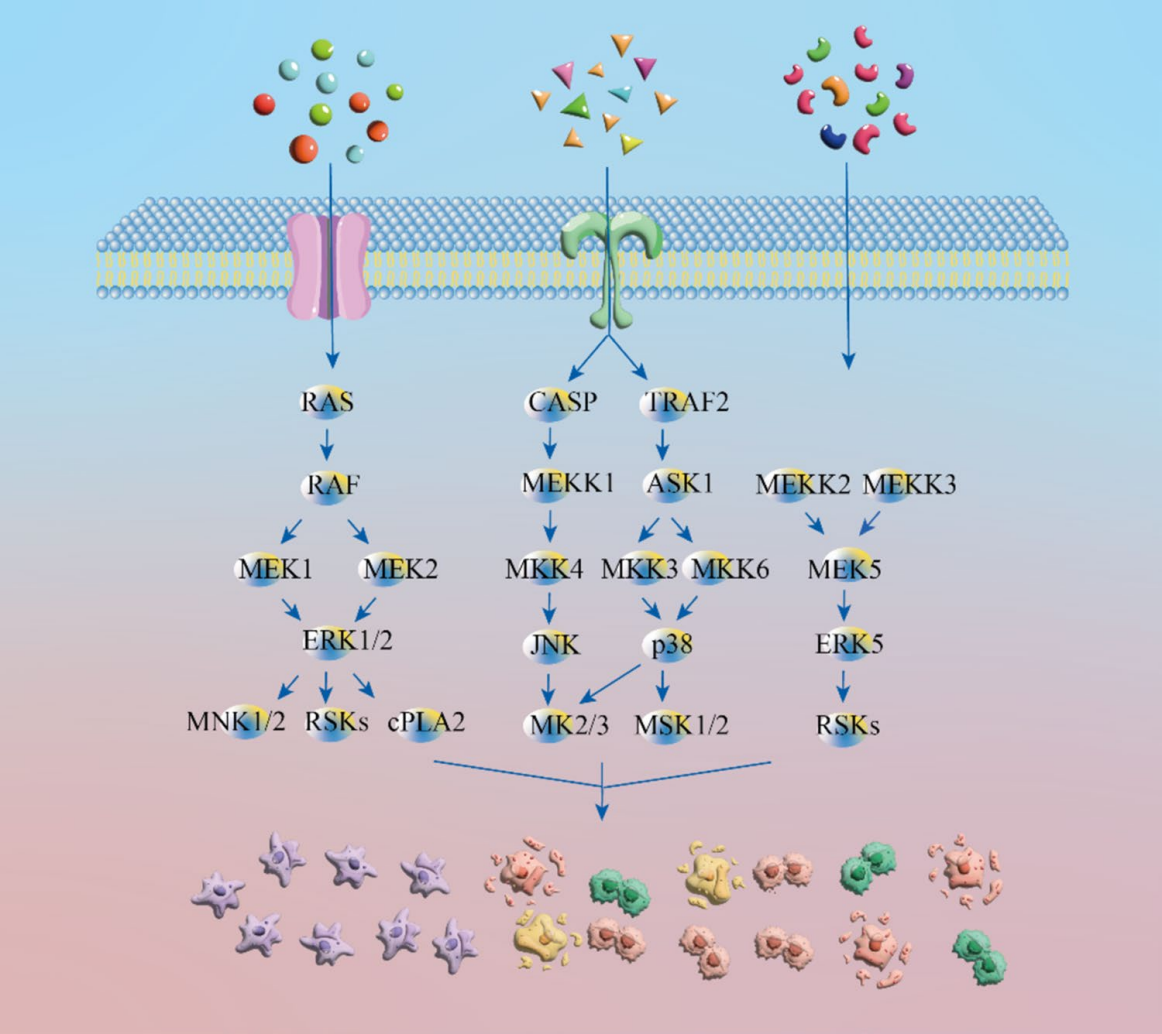
Three major transduction pathways of the MAPK-signaling pathway. When chemical molecules of natural products enter the cell membrane, they interact with key target proteins of the MAPK-signaling pathway. These in turn activate the cascade response of the MAPK-signaling pathway, thereby regulating apoptosis in cancer cells

ERK5 is another protein kinase of the triple MAPK-signaling cascade. It has three types: ERKa, ERKb, and ERKc. ERK5 has a C-terminal extension that includes a nuclear localization signal and a transcriptional transactivation domain (Cristea et al. 2020). Its activation is achieved through MEK5 phosphorylation. When its kinase domain is activated, ERK5 can phosphorylate multiple residues between its C-terminus. S753 and T732 have been characterized, and phosphorylation-specific antibodies have been produced against them (Cook and Lochhead 2022). ERK5 has diverse expression levels in various tissues, with high expression levels in the brain, thymus, and spleen. This signaling pathway participates in cell growth, differentiation, and decay and is related to cancer (Tubita et al. 2022). The three main transduction pathways of the MAPK-signaling pathway are shown in Fig. 1.

## MAPK in homeostasis

When regulating energy homeostasis, glycogen is usually the first choice for cells to store and use energy. Some research has shown that lipocytes scale up energy consumption by consistently expressing UCP1 in response to long-term sympathetic activation. ROS are produced during glycogen turnover. ROS can activate p38 MAPK, which drives the expression of UCP1. However, in the presence of an ROS scavenger, p38 activation and UCP1 expression levels were significantly decreased. While it remains unknown if ROS can directly activate p38, this work indirectly demonstrated that p38 is an essential link for regulating energy homeostasis (Keinan et al. 2021). Furthermore, the p38 MAPK pathway is also closely related to the maintenance of lipid homeostasis. In vitro experiments showed that hypothalamic ependymal–glia (tanycytes) can produce and secrete Fgf21 to regulate lipid homeostasis. Tanycytes can oxidize palmitate, produce ROS, and trigger the p38 MAPK pathway, which is critical for tanycytic Fgf21 expression upon palmitate exposure. When the p38 MAPK inhibitor SB203580 was used, the expression and secretion of palmitate-induced Fgf21 were prevented in cultured tanycytes. Thus, the p38 MAPK pathway is necessary for Fgf21 secretion and expression, highlighting its importance in lipid homeostasis (Geller et al. 2019). The MEK–ERK pathway is also related to cell homeostasis, with Feng et al. (2021) demonstrating that inhibition of excess ROS or the MEK/ERK pathway could save the survival of PDK1-deficient Treg cells.

The MAPK-signaling pathway is also involved in maintaining protein homeostasis. In the ECM, TMEM2 decomposes glycosaminoglycan HA and then changes ER stress resistance and stress sensitivity. The latter depends on the cell surface receptor CD44 and ERK and p38 pathways, which is mainly achieved through the ERK and p38 orthologues *MPK-1* and *PMK-1*. In addition, human studies and experiments of C. elegans prove that deletion of ERK or p38 inhibits the advantage of TMEM2 and that HTMEM2 relies on *PMK-1* to regulate immune homeostasis (Schinzel et al. 2019).

According to one study, AICD is a form of apoptosis. When the source of inflammation has been eliminated, the organism can use this process to shut down the T-cell response and then maintain lymphocyte homeostasis. This begins with the activation of JNK1 and ERK1/2 induced by AICD, which then leads to activation of the apoptogenic factor *BIM* and fission protein DRP1. In this process, ERK1/2 upregulates the *Bim-L* and *Bim-S* subtypes and cooperates with JNK1 to activate DRP1. JNK1 does not affect the activation of ERK1/2 during AICD. However, when mitochondrial structural alterations begin, ERK1/2 needs to maintain JNK1 activation via phosphorylation to maintain lymphatic T-cell homeostasis (Simula et al. 2020).

**Fig. 2:**
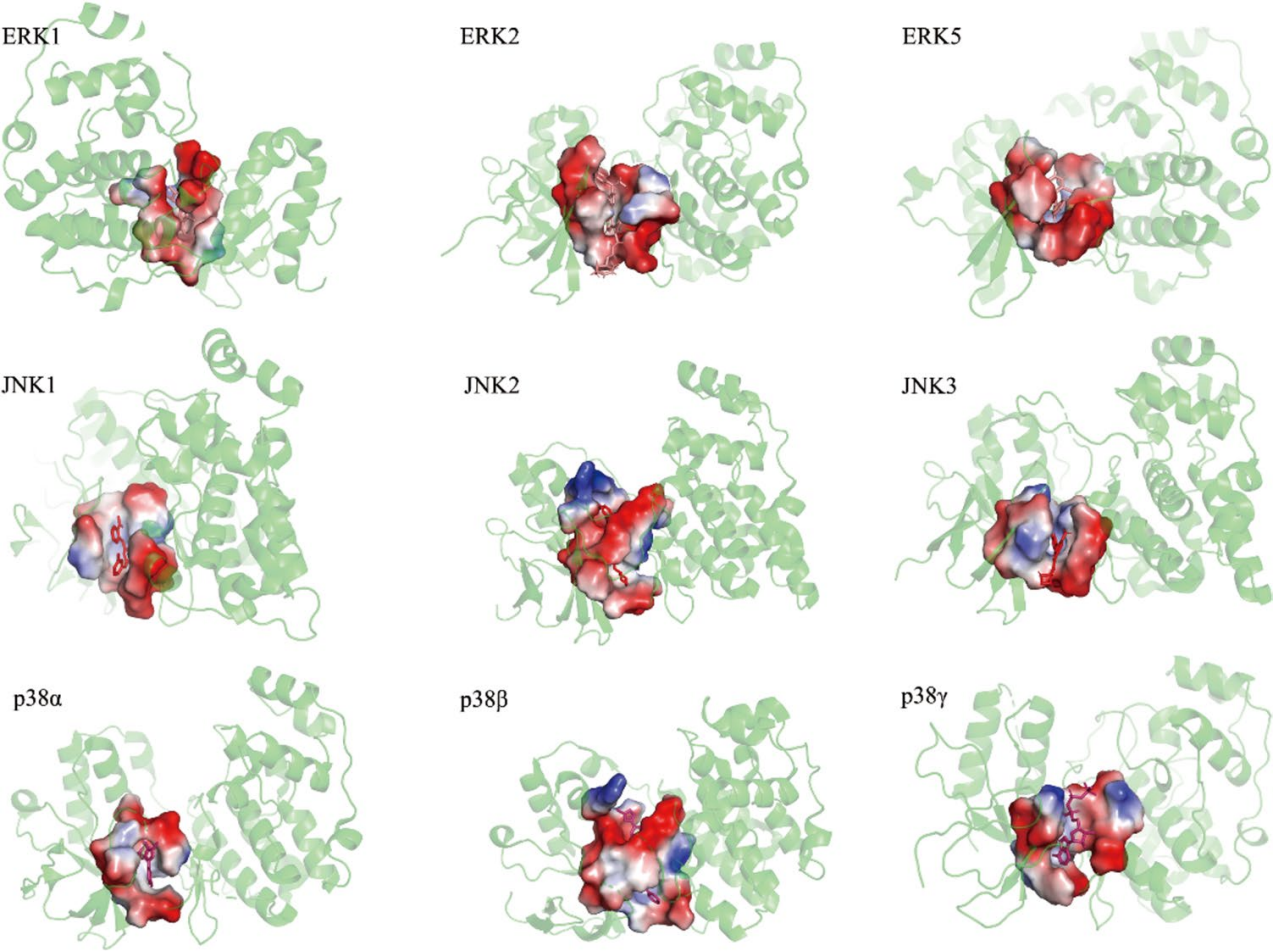
Main protein conformations and protein ligand pockets that affect homeostasis in the MAPK-signaling pathway

Tadokoro et al. (2018) discuss how diet affects HSC homeostasis, an animal model fed with a high-fat diet showed that, Spred1 can negatively regulate the RAS–MAPK-signaling pathway to protect HSC homeostasis. In addition, Spred1 can regulate HSC homeostasis through the ERK-signaling pathway, because the serum SCF levels will be increased from obesity. This excessive SCF can overactivate the ERK pathway, resulting in abnormal self-renewal of HSCs and maintenance of hemopoietic homeostasis. Furthermore, Drosophila ISCs play an essential role in gut homeostasis. If ISCs grow abnormally, gut homeostasis can be destroyed and cause a series of diseases, such as colon cancer. However, the Egfr–Ras85D/Ras1 MAPK-signaling pathway is the main element in ISC proliferation. When *SH3PX1*-dependent autophagy function is lost, Egfr will be surrounded by Rab11-endosomes at the cell surface. This hyperactivates the MAPK/ERK-signaling pathway and volitionally provokes ISC hyperplasia. In human cell experiments, it was also shown that inhibition of autophagy could increase ERK phosphorylation levels. Therefore, it is important to determine how the MAPK pathway maintains gut homeostasis for the treatment of gut diseases (Zhang et al. 2019a, b).The major protein kinases of the MAPK-signaling pathway and their protein–ligand pockets are shown in Fig. 2.

## MAPK-signaling pathway and cancer

MAPK signal transduction is an important pathway that adjusts cellular life processes and abnormal changes. Various MAPK pathway-related protein kinases have been found in cancer research, and their genetic modification is closely connected with cancer. Activation of this pathway can trigger microscopic tumor cell proliferation, differentiation, and apoptosis. For example, approximately 25% of mutations in human cancer are related to KRAS, but KRAS-targeted therapeutic agents are lacking. MEK and RAF1 in the MAPK-signaling pathway may be potentially critical targets for blocking signaling from mutated KRAS (Drosten and Barbacid 2020; Wang et al. 2019). Liu et al. (2022) clarified the connection between MAPK signaling and ovarian cancer cells, demonstrating that the activation of this pathway induced resistance to palbociclib. Stramucci et al. (2019) demonstrated that MKK3 can activate the p38 pathway to maintain survival signal transduction in colorectal cancer cells. The MAPK-signaling pathway is often triggered in various cancers and is considered a promising direction for cancer therapeutic development (Herman et al. 2022; Li et al. 2022; Ravichandran et al. 2022; Zhang et al. 2022).

Currently, there are seven drugs targeting the MAPK-signaling pathway for cancer treatment worldwide, including selumetinib sulfate, pirfenidone, cobimetinib, trametinib, binimetinib, donafenib tosylate, and tivozanib (Behr et al. 2021; Ascierto et al. 2023; Gershenson et al. 2022). Other cancer therapeutics related to MAPK signal transduction are in the early clinical trial development. Examples include the ERK1/2 inhibitor LY3214996, selumetinib, ulixertinib, fluorouracil, PDR001, bortezomib, and a combination of trametinib and dabrafenib (Gross et al. 2020; Jung et al. 2022; Bouffet et al. 2023). Other therapeutic agents in various clinical trial phases are shown in Table 1.

**Table 1:** Anti-cancer interventions that have impacted targeting the MAPK-signaling pathway at various clinical trial stages

Phase	ClinicalTrials.gov/NCTNumber	Interventions	Enrolment	Disease
1	NCT05557045	JZP815	320	Advanced cancer, metastatic cancer, solid tumor
1	NCT04959981	ERAS-007, ERAS-601, Osimertinib, Sotorasib	200	Advanced non-squamous non-small-cell lung cancer
1	NCT01392521	Copanlisib, Refametinib (BAY86-9766)	64	Neoplasms
1	NCT04418167	JSI-1187, Dabrafenib	124	Solid tumors
1	NCT05488821	QLH11906	40	Advanced solid tumors harboring mapk pathway alterations
1	NCT04495127	Selumetinib	12	Neurofibromatosis Type 1
1	NCT01668017	Pimasertib	26	Advanced solid tumors, hepatocellular carcinoma
1	NCT02313012	CC-90003	19	Neoplasm metastasis
1	NCT04081259	LY3214996	30	Acute myeloid leukemia
1	NCT02711345	LTT462	65	Ovarian neoplasms, non-small-cell lung, carcinoma, melanoma, other solid tumors
1	NCT02607813	LXH254, PDR001	142	NSCLC, ovarian cancer, melanoma, other solid tumors
1	NCT01463631	LY3007113	27	Metastatic cancer
1	NCT01393990	LY2228820, Midazolam, Tamoxifen	89	Advanced cancer
1	NCT04145297	Ulixertinib, Hydroxychloroquine	12	Gastrointestinal neoplasms
1	NCT01764828	Refametinib (BAY86-9766), Gemcitabine	23	Neoplasms
2	NCT04735068	Binimetinib Pill, Hydroxychloroquine Pill	29	Non-small cell lung cancer, KRAS mutation-related tumors
2	NCT03149029	Pembrolizumab, Dabrafenib, Trametinib	16	Metastatic melanoma
2	NCT05221320	Ulixertinib, Hydroxychloroquine	215	Tumor, solid, gastrointestinal cancer
2	NCT02625337	Pembrolizumab, Dabrafenib, Trametinib	32	Metastatic melanoma
2	NCT03363217	Trametinib	114	Low-grade glioma, plexiform neurofibroma, central nervous system glioma
2	NCT01160718	Fulvestrant, selumetinib	46	Breast cancer
2	NCT04534283	Abemaciclib, LY3214996	35	Cancer, cancer metastatic, BRAF V600E, MEK1 gene mutation, MEK2 gene mutation, ERK mutation, RAF1 gene mutation
2	NCT01229150	AZD6244, Erlotinib	89	Non-small cell lung carcinoma
1and 2	NCT01362803	AZD6244	99	Neurofibromatosis 1, Neurofibromatosis Type 1, NF1, Neurofibroma, Plexiform
1and 2	NCT02296242	BVD-523	53	Acute myelogenous leukemia, myelodysplastic syndrome
1and 2	NCT05578092	MRTX0902, MRTX849	225	Solid tumor, advanced solid tumor, non-small cell lung cancer, colo-rectal cancer
1and 2	NCT04892017	DCC-3116, Trametinib, Binimetinib, Sotorasib	323	Pancreatic ductal adenocarcinoma, non-small cell lung cancer, colorectal cancer, melanoma, advanced solid tumor, metastatic solid tumor
1and 2	NCT04985604	DAY101, Pimasertib, Hydrochloride	168	Melanoma, solid tumor
1and 2	NCT05054374	Mirdametinib, Fulvestrant	150	Breast cancer, breast cancer stage IV, HER2-negative breast cancer, solid carcinoma, MEK1 gene mutation, MEK2 gene mutation, metastatic breast cancer
1and 2	NCT05039177	ERAS-007, Encorafenib, Cetuximab, Palbociclib	200	Metastatic colorectal cancer, metastatic pancreatic ductal adenocarcinoma
1and 2	NCT01663857	LY2228820, Carboplatin, Placebo, Gemcitabine	118	Epithelial ovarian cancer, fallopian tube cancer, primary peritoneal cancer

### MAPK-signaling pathway in breast cancer

Breast cancer is a frequently observed tumor in women. While approximately 70–80% of early non-metastatic patients can be cured, advanced cases with organ metastasis are currently considered incurable. Metastatic factors are a major cause of patient death (Harbeck et al. 2019; Xu et al. 2020). Breast cancer is a genetically heterogeneous disease, with six basic molecular subtypes. Nulliparity, having fewer children, and later age at menopause are all related to an increased risk of breast cancer (Pashayan et al. 2020; Pedrosa et al. 2018).

Wen et al. (2019) suggested that the MAPK-signaling pathway may be a key target for treating breast cancer. Cancer-associated fibroblast-derived IL-32 specifically combines with integrin β3 via the RGD motif, then activates the p38 pathway. This upregulates the expression levels of epithelial–mesenchymal transition markers and promotes cancer cell invasion (Wen et al. 2019; Elwakeel et al. 2022). Another study explored the mechanism of the IncRNA prncr1 and MAPK-signaling pathway in breast cancer. The study emphasized that this lncRNA competes with miR-377, which causes an increase in *CCND2* expression levels. The MEK protein kinase can then be activated and support tumor cell growth (Ouyang et al. 2021).

Sequencing technologies have helped clarify many genomic changes that occur in breast cancer, such as mutations in *FOXA1*, *PIK3CA*, *ERBB2*, and *SPOP E78K*. These may serve as transfer or treatment functions. Among these mutations, *ERBB2* is a hotspot mutation in breast cancer that can induce RAS/RAF/MAPK-signaling pathway activity, and the *NF1* deletion mutation is also involved in MAPK activation (Razavi et al. 2018). Clinical data have shown that some MAPK-signaling pathway inhibitors are being studied. For example, mirametinib is used alone or combined with fulvestrant. However, the research results of certain drugs are not satisfactory. For instance, the results of the combination of fulvestrant and selumetinib suggested that this approach did not improve patient prognosis. In addition, selumetinib may reduce the effectiveness of endocrine therapy, and there is also a problem of poor tolerance with monotherapy doses (Zaman et al. 2015).

### MAPK-signaling pathway in colorectal cancer

The incidence and mortality rate of colorectal cancer are increasing every year. The standard treatments for this disease are chemotherapy, radiotherapy, and surgery (Johdi and Sukor 2020). However, the recurrence rate of colorectal cancer is high. After radical resection of the primary tumor, approximately 30–40% of colorectal cancer patients will develop metastases. Therefore, a therapeutic method to prevent recurrence is needed (Cañellas-Socias et al. 2022).

Changes in the MAPK-signaling pathway are common in colorectal cancer. For example, through animal experiments, Bai et al. (2022) showed that smoke can induce gut microbiota dysbiosis and damage the intestinal shielding function, which may activate the carcinogenic ERK/MAPK pathway in the colon epithelium. In addition, ubiquitin-specific protease can accelerate the proliferation and growth of colorectal cancer cells via the ERK/MAPK pathway by stabilizing protein phosphatase 1 catalytic subunit alpha (Sun et al. 2019).

Most colorectal cancer cells depend on EGFR/KRAS/BRAF/MAPK-signaling pathway activation (Johnson et al. 2022). However, in KRAS and BRAF-mutant colorectal tumors, direct targeting of the MAPK-signaling pathway to inhibit ERK activation has been clinically unsuccessful, but the use of a combination approach that included EGFR inhibition has achieved promising results (Ponsioen et al. 2021). Recently, EGFR inhibitors, such as cetuximab, leucovorin, and oxaliplatin, have been used to treat colorectal cancer (Parseghian et al. 2023; Elez et al. 2022). In colorectal cancer, RAF is the most commonly mutated gene in this pathway. This occurs in approximately 10% of patients with metastatic colorectal cancer (Grothey et al. 2021). However, according to clinical research, only approximately half of cancer patients respond to single-drug treatment with BRAF inhibitors. This is because the adaptive feedback of the MAPK pathway mediated by EGFR is reactivated. Therefore, targeting this adaptive feedback pathway in such colorectal cancer cases can enhance the curative effect. However, MAPK reactivation remains a key issue with primary and acquired drug resistance (Corcoran et al. 2018).

### MAPK-signaling pathway in pancreatic cancer

Hereditary factors are a main contributor to pancreatic cancer development. The incidence of pancreatic cancer is high in North America and Europe, and smoking, alcohol consumption, and a high cholesterol diet may all contribute to the risk of developing pancreatic cancer. Pancreatic cancer patients are usually categorized into resectable, marginally resectable, locally advanced, and metastatic groups according to the degree of disease. Surgical resection is currently the main treatment method. Systemic chemotherapy combinations are often administered to patients with advanced disease (Mizrahi et al. 2020).

The microenvironment of pancreatic cancer has received increasing attention and consists mainly of cancer cells, stromal cells and extracellular components (Ren et al. 2018a, b). PDAC is the most commonly observed intraepithelial tumor of the pancreas (Vincent et al. 2011). Bryant et al. (2019) suggested that PDAC characteristics include KRAS and autophagy-dependent tumor growth, demonstrating that the inhibition of KRAS and ERK could increase autophagic flow. From their data, the authors believe that a drug combination that could simultaneously inhibit ERK and upregulate the autophagy process would be a possibly effective therapeutic approach. Ravichandran et al. (2022) treated PDAC with the MEK inhibitor trimetinib. MAPK-signaling pathway inhibition resulted in decreased c-MYC expression levels and an increase in MiT/TFE-dependent lysosomal biogenesis. The destruction of ferritinophagy synergizes and cooperates with the KRAS/MAPK-signaling pathway to inhibit PDAC growth, highlighting a key target of metabolic dependency.

In another study, Lin et al. (2021) also showed that the MAPK pathway is connected to pancreatic cancer cells. PA cells were significantly inhibited when a PKC/MEK inhibitor was added to cancer cells overexpressing TRPM2. The results suggested that TRPM2 possibly directly activates PKCα through calcium or indirectly triggers PKCε and PKCδ through increased DAG. This then activates the MAPK-signaling pathway to promote PA growth.

Pancreatic cancer is frequently at an advanced stage when diagnosed (Klein 2021) and has developed chemotherapy resistance, which contributes to the unsatisfactory treatment of pancreatic cancer patients. Targeting the MAPK-signaling pathway may provide a new option for treating pancreatic cancer.

### MAPK-signaling pathway in gastric cancer

Gastric cancer is a highly molecularly and phenotypically heterogeneous disease. Helicobacter pylori infection, pickled food, and smoking are all risk factors for gastric cancer (Smyth et al. 2020). Differences in tumor biology between Eastern and Western countries increase the complexity of international standard treatments. The effective ways to treat gastric cancer include systemic chemotherapy, immunotherapy, surgery, targeted therapy, and radiotherapy. Drugs approved for the treatment of gastric cancer include ramucirumab and pembrolizumab (Joshi and Badgwell 2021).

The MAPK-signaling pathway is connected with multiple factors in gastric cancer development, metastasis, and treatment. The secondary messenger Ca^2+^ is a crucial regulatory factor during gastric cancer metastasis. Calcium release activates the calcium regulator (ORAI2), which can enhance cancer cell metastasis by inducing FAK-mediated MAPK/ERK pathway activation (Wu et al. 2021). HDACs are a hot topic, with inhibition of HDACs being recognized as a cancer treatment method. Functional measurements showed that Class IIA (*HDAC4*) is upregulated in gastric cancer cells and related to poor prognosis. *HDAC4* inhibits the transcription of *ATG4B* in an *MEF2A*-driven manner, prevents *MEKK3* from p62-dependent autophagic degradation, and then activates p38 protein kinase. *HDAC4* plays a carcinogenic role in gastric cancer. Targeted treatment focused on *HDAC4* may be a new strategy (Zang et al. 2022).

One report described a new non-coding RNA, circMAPK1, that is involved in the MAPK-signaling pathway, with its expression levels decreasing in gastric cancer. However, lower circMAPK1 expression levels are associated with lower survival rates in cancer patients. In gastric cancer, circMAPK1 plays an inhibitory role by encoding the MAPK1-109aa protein, specifically by inhibiting the phosphorylation of MAPK1 by competitively combining with MEK1 and then repressing the downstream MAPK pathway. CircMAPK1 is a good predictor of gastric cancer and provides a direction for treatment (Jiang et al. 2021).

There are many studies on gastric cancer cases involving human EGFR2. For example, in a clinical phase III study for advanced HER2 cancer, adding pembrolizumab to chemotherapy and trastuzumab could significantly reduce tumor size and improve the objective remission rates (Janjigian et al. 2021). However, because of the abnormal activation of *HER2* and downstream signals, such as the amplification, mutation, or upregulation of *HER2*, *KRAS*, and *AKT*, the sensitivity and drug resistance of patients are insufficient. Poor patient response remains a clinical challenge (Shi et al. 2021). Inhibitors of the MAPK-signaling pathway have good efficacy when combined with other drugs, but some of these drugs alone have poor efficacy. For example, the MEK1 gene can have an activation mutation that causes gastric cancer. After treatment with trametinib alone, ERK1/2 is reactivated and the cancer cells become resistant to the drug. However, when used in combination with lapatinib, ERK1/2 activation was reversed and drug resistance was eliminated. Therefore, using drugs in combination is a possible treatment method to overcome drug resistance (Mizukami et al. 2015; Wang et al. 2021a, b).The anti-cancer intervention measures that affect the targeted MAPK-signaling pathway at different stages of clinical trials are shown in Table 1.

## Natural product-targeted MAPK-signaling pathway for cancer prevention and treatment

Natural products play a significant role in treating diseases, especially various cancers and contagions, and as such have elicited the attention of many researchers (Atanasov et al. 2021). The chemical composition of products from different sources has become a promising method for preventing and treating cancer. A series of natural products are related to many signaling pathways and play an anti-tumor role. For example, sulforaphane, curcumin, quercetin, and resveratrol can affect the MAPK, PI3K/Akt, NF-κB, and other pathways to regulate the growth and proliferation of cancer cells (Shakeri et al. 2021). The following sections describe some of the natural active ingredients, summarize the relevant literature on natural products targeting the MAPK-signaling pathway in cancer, present preclinical studies from the last 5 years on different cancer types, and elucidate their mechanisms of action.

### Flavonoids

**Fig. 3:**
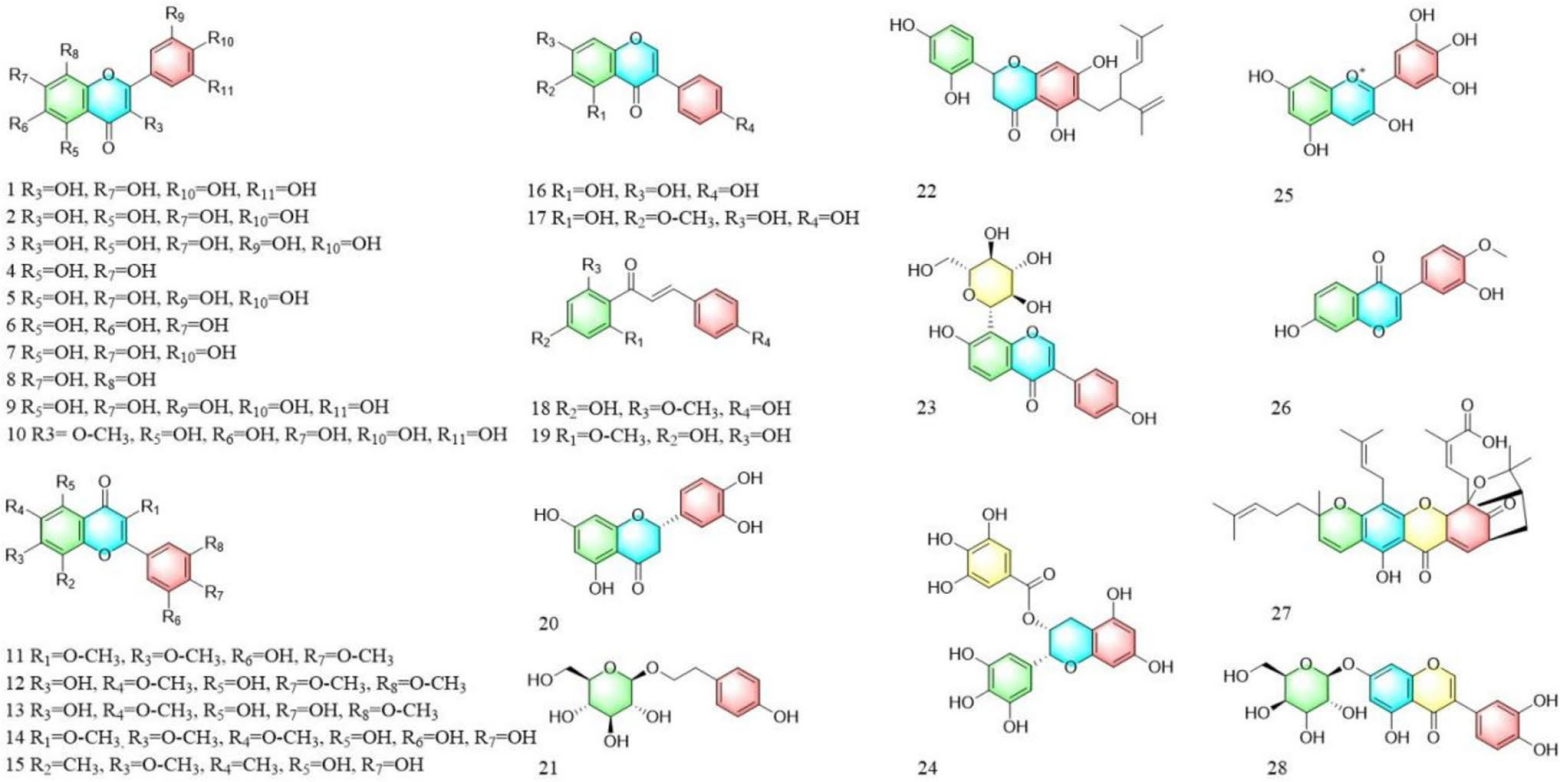
Structures of 28 flavonoids

Flavonoids belong to plant secondary metabolites, a class of compounds with C6–C3–C6 as their basic skeleton. In plants, this is mainly bound to sugars in the form of glycosides or carboglycosyl groups, and to a lesser extent in free form (Imran et al. 2019). Among the natural products that have been discovered so far, flavonoid components have been demonstrated to have significant anti-tumor activity by numerous studies. For example, eupatilin (Wang et al. 2018a, b, c, d) is a natural flavonoid. In esophageal tumor cells, eupatilin can inhibit ERK1/2 phosphorylation and growth of the esophageal tumor cell line TE1. In trials with the endometrial cancer cell lines Hec1A and KLE, eupatilin could upregulate ERK1/2 phosphorylation and inhibit tumor cell proliferation. Compared with cisplatin, its inhibitory effect was more effective and had fewer associated toxicity and side effects. According to reports, the activity of ERK1/2 protein is also related to apoptosis of oral squamous cell carcinoma cells. Hsieh et al. (2021) found that chrysosplenol D could inhibit the activation of key target proteins in the MAPK-signaling pathway, including ERK1/2, JNK, and p38, thereby enhancing the cleaved PARP activation and mediating the arrest and apoptosis of cancer cells. Flavonoids targeting the MAPK-signaling pathway and their regulatory mechanisms are shown in Table 2 and the flavonoid structures are shown in Fig. 3.

**Table 2:** Detailed information of 28 flavonoids

No	Ingredients	Diseases	Use of cell lines	Mechanism of action	References
1	Fisetin	Non-small cell lung cancer	NCI-H460	↑p-ERK,↑p-JNK and ↑p–p38	Chen and Liu (2018)
2	Kaempferol	Oophoroma	OVCAR-3 and A2780/CP70	↓p-ERK	Chen and Liu (2018)
3	Quercetin	Liver cancer	HepG2	↑p-JNK, ↑p–p38,↓p-ERK1/2 and↓MEK1	Ding et al. (2018)
4	Chrysin	Oophoroma	ES2 and OV90	↑ERK1/2,↑JNK and↑p38	Lim et al. (2018a, b)
5	Luteolin	Cervical carcinoma	HeLa	↓p-ERK and ↓p–p38	Raina et al. (2021)
6	Baicalein	Colon cancer	HCT116,A549 and panc-1	↑p-ERK,↑p-JNK and↑p–p38	Su et al. (2018)
7	Apigenin	Bladder cancer	T24	↓p-ERK1/2 and ↓p-JNK	Xia et al. (2018)
8	7,8-dihydroxyflavone	Osteosarcoma	U2OS and 143B	↓p–p38,↑p-JNK,↑p-ERK1/2	Zhao et al. (2021a, b)
9	Myricetin	Osteosarcoma	D-17 and DSN	↑p-JNK and↑p-ERK1/2	Park et al. (2018b, a)
10	Quercetin-3-methyl ether	Esophageal squamous cell carcinoma	KYSE450 and KYSE510	↓p-ERKs	Zhao et al. (2018)
11	3,4',7-O-trimethylquercetin	Ovarian cancer	CRL-1978	↓p38	Ashraf et al. (2018)
12	Eupatilin	Esophageal cancer	TE1	↓p-ERK1/2	Wang et al. (2018a, b, c, d)
13	Jaceosidin	Breast cancer	MCF-7	↑p-ERK and↑p–p38	Ojulari et al. (2021)
14	Chrysosplenol D	Oral squamous cell carcinoma	SCC-9 and HSC-3	↓p-ERK1/2,↓p-JNK1/2 and ↓p–p38	Hsieh et al. (2021)
15	Sideroxylin	Oophoroma	ES2 and OV90	↑p-ERK1/2,↑p-JNK and↑p–p38	Park et al. (2018b, a)
16	Genistein	Non-small cell lung cancer	NCI-H460	↑p-ERK,↑p-JNK and↑p–p38	Chen and Liu (2018)
17	Tectorigenin	Breast cancer	MDA-MB-231 and MCF-7	↓p-ERK, ↓p-JNK and ↓p–p38	Yang et al. (2021)
18	3-deoxysappanchalcone	Esophageal squamous cell carcinoma	KYSE 30/70/410/450/510	↑p-JNK and↑p–p38	Kwak et al. (2021)
19	Cardamonin	Osteosarcoma	OS	↑p-JNK and↑p–p38	Zhang et al. (2021a, b, c)
20	Eriodictyol	Glioblastoma	A172 and U87 MG	↓p–p38	Lv et al. (2021)
21	Slaidroside	Colorectal cancer	HT29	↑JNK and↑p38	El-Kott et al. (2021)
22	Sophoraflavanone G	Triple negative breast cancer	MDA-MB-231	↓p-ERK1/2,↓p-JNK and ↓p–p38	Yang et al. (2021)
23	Puerarin	Non-small cell lung cancer	NSCLC	↓p-ERK	Hu et al. (2018)
24	(-)-epigallocatechin gallate	Triple negative breast cancer	MDA-MB-231	↓p-ERK	Huang et al. (2021a, b)
25	Delphinidin	Melanoma	HOS and MG-63	↓p-ERK and ↓p–p38	Kang et al. (2018)
26	Calycosin	Gastric cancer	AGS	↑p-JNK, ↑p–p38 and ↓p-ERK	Zhang et al. (2021a, b, c)
27	Gambogic Acid	Prostatic cancer	PTEN-//p53-/PC and LAPC-4	↓ERK1/2 and↓p-MEK1/2	Pan et al. (2018)
28	Luteoloside	Cervical carcinoma	Hela	↑p-JNK and↑p–p38	Shao et al. (2018)

### Phenols

**Fig. 4:**
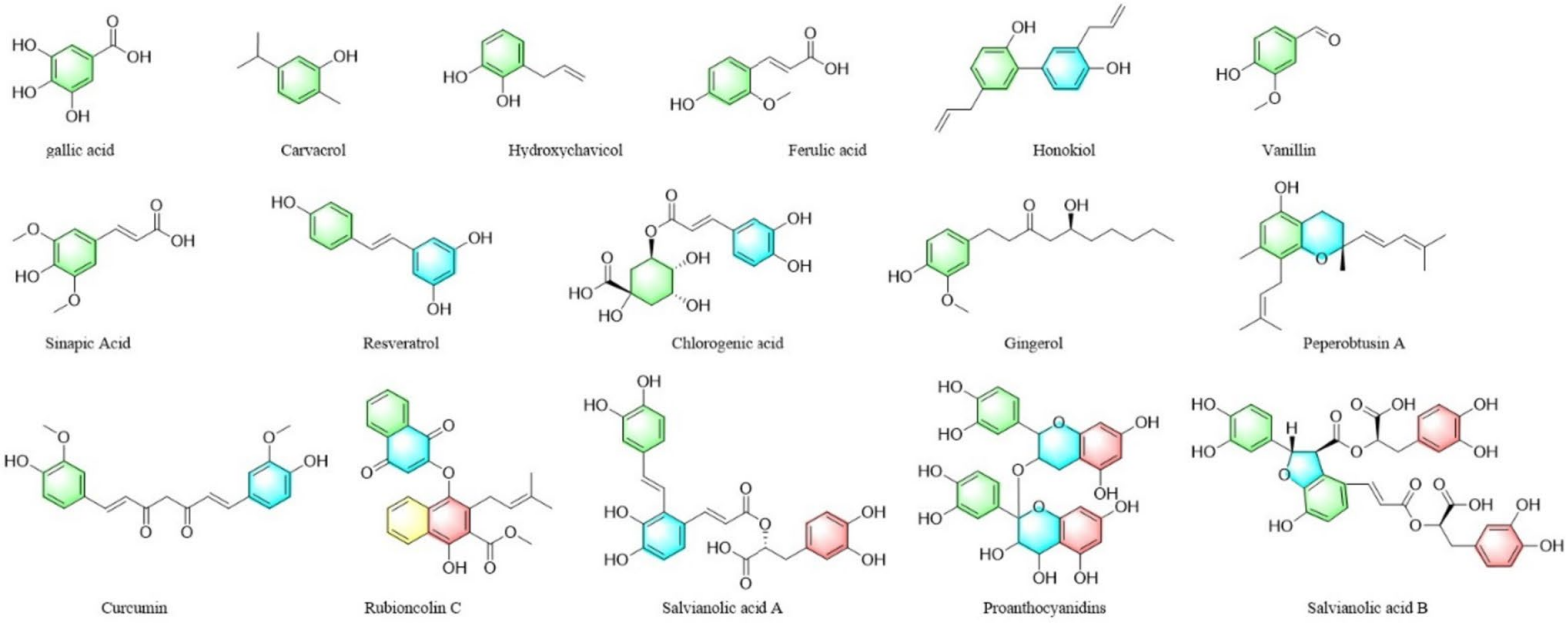
Structures of 16 phenols

Phenolic compounds are plant secondary metabolites that are widely found in nature. Phenolic compounds are structurally diverse and have antioxidant, anti-tumor, and antiviral effects (Almanza-Aguilera et al. 2023; Huminiecki 2022; Sorrenti et al. 2023). Phenolic compounds can regulate cancer cell apoptosis by modulating protein kinases in the MAPK-signaling pathway. Vanillin is a natural aromatic organic compound mainly found in plants, including vanilla pompon, vanilla planifolia, and vanilla tahitiensis. Vanillin can upregulate p38 phosphorylation in colorectal cancer cells and increase cancer cell apoptosis, with almost no treatment-related side effects (Li et al. 2021a, b, c). According to another study, vanillin can also inhibit the MAPKsignaling pathway, downregulate the phosphorylation of ERK, JNK, and p38 protein kinases, and reduce the number of cancer cells in colon tissues (Li et al. 2018a, b). Resveratrol is a natural phenolic compound with high anti-cancer activity and can be used to treat various cancers. According to a previous study, resveratrol can inhibit the ERK and p38 MAPK-signaling pathways by downregulating ERK and p38 phosphorylation in pancreatic cancer cells, reducing the proliferation and diffusion of cancer cells (Chen and Liu 2018). In addition, resveratrol prevents interleukin-6-induced gastric cancer metastasis by inhibiting RAF/MAPK pathway activation (Yang et al. 2018). The anti-cancer mechanisms of action of phenolic compounds are shown in Table 3 and the structures are illustrated in Fig. 4.

**Table 3:** Detailed information of 16 phenols

No	Ingredients	Diseases	Use of cell lines	Mechanism of action	References
1	Gallic acid	Bladder cancer	TSGH-8301	↓p-ERK	Liao et al. (2018)
2	Carvacrol	Choriocarcinoma	JAR and JEG3	↓p-ERK1/2,↑p-JNK and↑p–p38	Lim et al. (2019)
3	Hydroxychavicol	Colon cancer	HT-29	↑p-JNK and↑p–p38	Rajedadram et al. (2021)
4	Ferulic acid	Lung cancer and Liver cancer	A549 and HepG2	↓p–p38	Das et al. (2019)
5	Honokiol	Oral squamous cell carcinoma	OC2 and OCSL	↓p-ERK, ↓p-JNK and ↓p–p38	Huang et al. (2018)
6	Vanillin	Colorectal cancer	SW480 and HT-29	↑p–p38	Li et al. (2021a, b, c)
		Colon cancer	HCT-116	↓p-ERK, ↓p-JNK and ↓p–p38	Li et al. (2018a, b)
7	Sinapic Acid	Human neuroblastoma	SH-SY6Y	↓p-ERK1/2,↓p-JNK and ↓p–p38	Tungalag and Yang (2021)
8	Resveratrol	Pancreatic cancer	Panc-1	↓p-ERK and ↓p–p38	Chen and Liu (2018)
		Gastric cancer	GC7901	↓RAF	Yang et al. (2018)
9	Chlorogenic acid	Lung cancer	A549	↑p38	Yamagata et al. (2018)
10	Gingerol	Colon cancer	HCT116	↑p-ERK,↑p-JNK and↑p–p38	Chen and Liu (2018)
11	Peperobtusin A	Lymphoma	U937	↑p–p38	Shi et al. (2018)
12	Curcumin	Retinoblastoma	RB Y79	↑p-JNK and↑p–p38	Wang et al. (2021a, b)
		Adrenocortical carcinoma	SW-13 and NCI-H295R	↑p-JNK and↑p–p38	Huang et al. (2021a, b)
13	Rubioncolin C	Triple negative breast cancer	MDA-MB-231	↑p-ERK,↑p-JNK and↑p–p38	Li et al. (2021a, b, c)
14	Salvianolic acid A	B-cell lymphoma	Raji and Jeko-1	↑p-JNK, ↓p-ERK and ↓p–p38	Li et al. (2021a, b, c)
15	Proanthocyanidins	Lung cancer	LLC and A549	↑p-JNK and↑p–p38	Xu et al. (2021)
16	Salvianolic acid B	Osteosarcoma	MG63	↑p–p38	Zeng et al. (2018a, b)

### Terpenoids

**Fig. 5:**
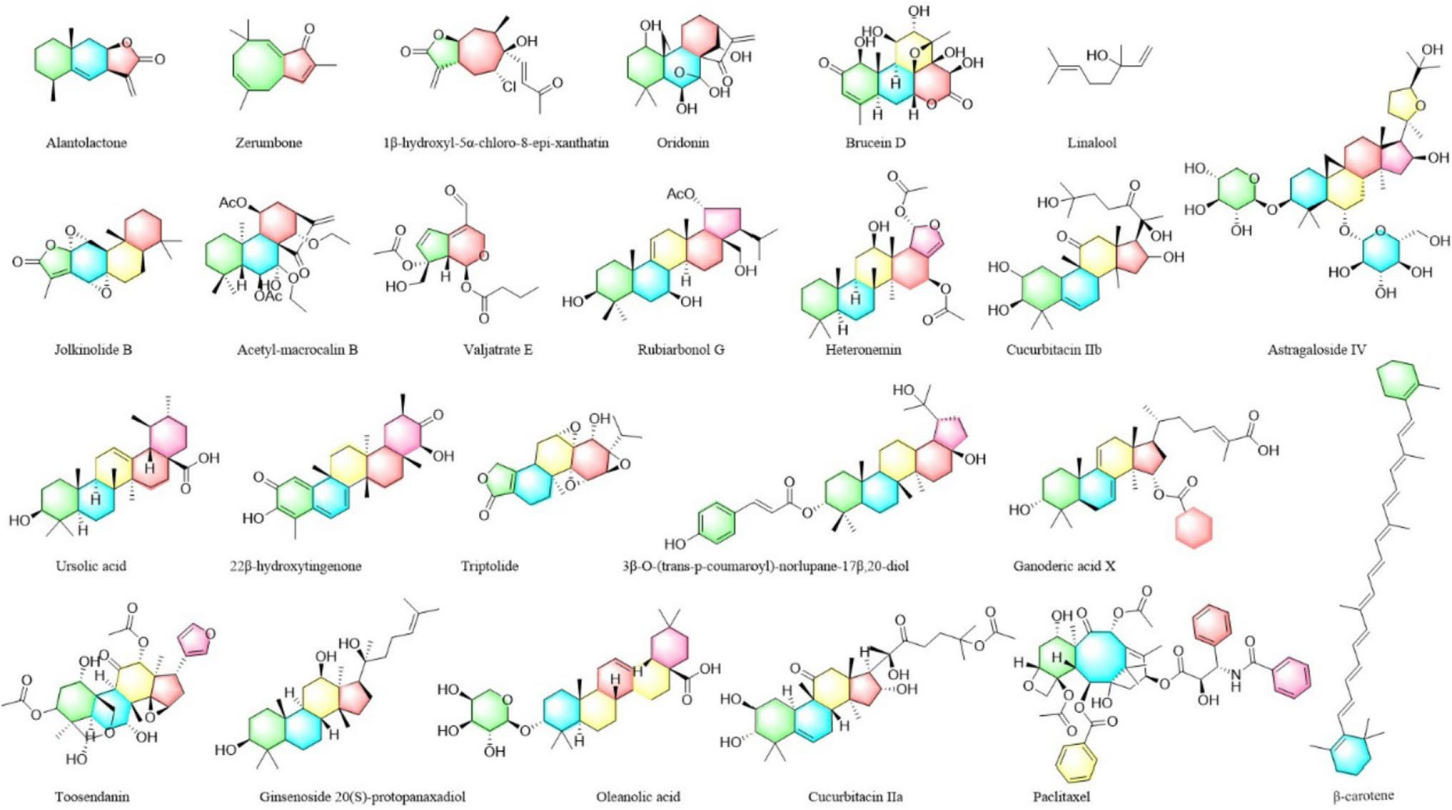
Structures of 24 terpenoids

Terpenes, most of which are polymers of isoprene and their derivatives, are a widely distributed class of natural products. They are classified based on the number of isoprene units in the molecular structure, including monoterpenes, sesquiterpenes, diterpenes, triterpenes, and tetraterpenes. Each isoform has different biological activities, with some having anti-cancer effects (Wei et al. 2023; Zhang et al. 2023). Zerumbone (Lv et al. 2018) is a monocyclic sesquiterpenoid compound that is isolated from the root of Zingiber Zerumbet Smith. In HepG2 liver cancer cells, zerumbone can inhibit the metastasis and proliferation of hepatoma cells in a dose-dependent manner by downregulating ERK1/2 phosphorylation and upregulating p38 phosphorylation. Jalili-Nik et al. (2021) also showed that zerumbone can suppress glioblastoma multiform cancer cell metastasis by reducing ERK1/2 phosphorylation. Oridonin is a natural tetracyclic diterpenoid compound. Oridonin may induce oral cancer cell apoptosis by the ROS-mediated p38 and JNK pathways (Oh et al. 2018). Research in colon and pancreatic cancers suggested that oridonin can significantly upregulate p38 phosphorylation and participate in cancer cell apoptosis. When using a p38-specific inhibitor (SB203580), inhibition of p38 significantly attenuated the increase in p–p53 levels induced by oridonin. These results showed that oridonin can trigger p53 signaling in cancer cells via the p38 pathway and was directly involved in cancer cell apoptosis (Liu et al. 2018; Chen and Liu 2018). The mechanisms by which terpenoids modulate the MAPK-signaling pathway are shown in Table 4 and the structures are shown in Fig. 5.

**Table 4:** Detailed information of 24 terpenoids

No	Ingredients	Diseases	Use of cell lines	Mechanism of action	References
1	Alantolactone	Breast cancer	MDA-MB-231	↑p-ERK, ↑p-JNK and↑p–p38	Cui et al. (2018)
2	Zerumbone	Glioblastoma multiforme	U-87 MG	↓ERK1/2	Jalili-Nik et al. (2021)
		Liver cancer	HepG2	↓p-ERK1/2 and↑p–p38	Lv et al. (2018)
3	1β-hydroxyl-5α-chloro-8-Epi-xanthatin	Liver cancer	HCC	↑p-ERK and↑p–p38	Fang et al. (2018)
4	Oridonin	Pancreatic cancer	SW1990	↑p–p38	Chen and Liu (2018)
		Oral squamous cell carcinoma	HN22 and HSC4	↑p-JNK and↑p–p38	Oh et al. (2018)
		Colon cancer	SW620	↑p–p38	Liu et al. (2018)
5	Brucein D	Breast cancer	MDA-MB-231 and MCF-7	↑p-JNK and↑p–p38	Mohan et al. (2021)
6	Linalool	Liver cancer	HepG2	↑p-ERK, ↑p-JNK and↑p–p38	Rodenak-Kladniew et al. (2018)
7	Jolkinolide B	Bladder cancer	T24 and UM-UC-3	↑p-ERK, ↑p-JNK and↑p–p38	Sang et al. (2021)
8	Acetyl-macrocalin B	Non-small cell lung cancer	A549 and H1299	↑p–p38	Wang et al. (2018a, b, c, d)
9	Valjatrate E	Liver cancer	HepG2	↓p-ERK	Sun et al. (2018)
10	Rubiarbonol G	Cervical carcinoma	HeLa	↑p-ERK1/2 and ↑p-JNK	Zeng et al. (2018a, b)
11	Heteronemin	Liver cancer	HA22T and HA59T	↓ERK and ↑JNK	Chang et al. (2021)
12	Cucurbitacin IIb	Liver cancer	A549	↓BRAF,↓Raf1,↓MEK1/2 and↓ERK2	Liang et al. (2021)
13	Astragaloside IV	Breast cancer	MDA-MB-231	↓p-ERK1/2	Chen et al. (2021)
		Glioma	U251	↓p-ERK1/2 and p-MEK	Chen et al. (2021)
		Cervical carcinoma	SiHa	↓p–p38	Chen et al. (2021)
		Breast cancer	MCF-7和MDA-MB-231	↓p-ERK and ↓p-JNK	Chen et al. (2021)
14	Ursolic acid	Breast cancer	MCF-7,MDA-MB-231 and SK-BR-3	↓p-ERK1/2	Chen and Liu (2018)
		Colon cancer	HT-29	↓p-ERK1/2,↓p-JNK and ↓p–p38	Chen and Liu (2018)
		Cervical carcinoma	HeLa	↓p-ERK1/2 and ↓p–p38	Chen and Liu (2018)
		Osteosarcoma	MG-63	↑p-ERK1/2,↑p-JNK and↑p–p38	Chen and Liu (2018)
15	22β-hydroxytingenone	Melanoma	SK-MEL-28	↓BRAF,↓NRAS and ↓KRAS	Aranha et al. (2021)
16	Triptolide	Cervical carcinoma	SiHa	↑p–p38	Qin et al. (2018)
17	3β-O-(trans-p-coumaroyl)-norlupane-17β,20-diol	Liver cancer	HepG2	↑p-ERK1/2,↑p-JNK and↑p–p38	Qi et al. (2021)
18	Ganoderic acid X	Liver cancer	HuH-7	↑p-ERK and ↑p-JNK	Gill et al. (2018)
19	Toosendanin	Gastric cancer	AGS and HGC-27	↑p–p38	Zhou et al. (2018)
20	Ginsenoside 20(S)-protopanaxadiol	Triple negative breast cancer	MDA-MB-231	↓ERK1/2,↓JNK and↓p38	Yang et al. (2021)
21	Oleanolic acid	Liver cancer	HepG2	↑p-ERK	Chen and Liu (2018)
22	Cucurbitacin IIa	Liver cancer	A549	↑p-BRAF,↓p-Raf1 and ↓p-MEK	Zhang et al. (2019a, b)
23	Paclitaxel	Breast cancer	CHMm	↑p–p38	Ren et al. (2018a, b)
24	β-carotene	Gastric adenocarcinoma	AGS	↓p-ERK1/2,↓p-JNK1/2 and ↓p–p38	Bae et al. (2021)

### Alkaloids

**Fig. 6:**
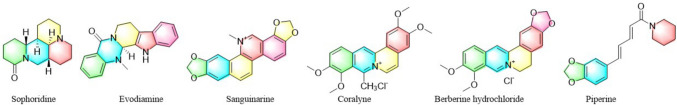
Structures of 6 alkaloids

Alkaloids are a class of basic nitrogen-containing organic compounds found in nature, most of which have complex circular structures. Alkaloids are one of the clinically effective ingredients in the treatment of diseases (Gjorgieva Ackova et al. 2023; Guo et al. 2023). Wang et al. (2018a, b, c, d) found that evodiamine could upregulate the phosphorylation levels of p38 and JNK in glioblastoma multiforme cells, which led cancer cell apoptosis. Evodiamine also induced apoptosis in ovarian cancer cells by disrupting the mitochondrial membrane potential through activation of JNK and ERK. Zhao et al. (2021a, b) found that sophoridine significantly upregulated the phosphorylation levels of JNK, ERK, and p38, which in turn promoted macrophage M1 polarization, thereby inhibiting cancer cell growth. The anti-cancer mechanisms of action of alkaloids are shown in Table 5 and the structures are illustrated in Fig. 6.

**Table 5:** Detailed information of 6 alkaloids

No	Ingredients	Diseases	Use of cell lines	Mechanism of action	References
1	Sophoridine	Non-small cell lung cancer	H460 and Lewis	↑p-ERK,↑p-JNK and↑p–p38	Zhao et al. (2021a, b)
2	Evodiamine	Glioblastoma multiforme	U251 and LN229	↑p-JNK and↑p–p38	Wang et al. (2018a, b, c, d)
3	Sanguinarine	Breast cancer	T47D and MDA-MB-231	↓p-ERK	Su et al. (2021)
4	Coralyne	Skin cancer	A431	↑p–p38	Bhattacharyya et al. (2018)
5	Berberine hydrochloride	Gastric cancer	AGS	↓p-ERK1/2 and ↓p–p38	Chen et al. (2018a, b)
6	Piperine	Oophoroma	A2780	↓p-JNK and ↓p–p38	Si et al. (2018)

### Steroidal saponins

**Fig. 7:**
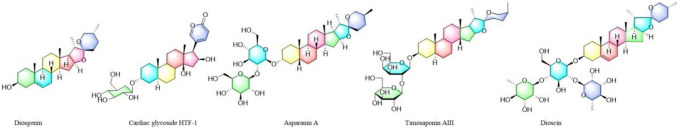
Structures of 5 steroidal saponins

Steroids are a wide class of chemical compounds in nature, all of which have a cyclopentano-perhydrophenanthrene parent nucleus in their structures. Certain steroidal saponins are already being used in cancer treatment studies (Bouabdallah et al. 2023; Majnooni et al. 2023). For example, timosaponin AIII is a steroid saponin that can play an anti-cancer role in different cancers, especially breast cancer. Work in MDA-MB-2 and MCF231 breast cancer cell lines suggested that timosaponin AIII could trigger DNA damage by activating p38, then indirectly cause G2/M phase arrest that reduced cell survival (Zhang et al. 2020). The mechanisms by which steroids can regulate the MAPK-signaling pathway are shown in Table 6 and their structures are shown in Fig. 7.

**Table 6:** Detailed information of 5 steroidal saponins

No	Ingredients	Diseases	Use of cell lines	Mechanism of action	References
1	Diosgenin	Oophoroma	SKOV3	↓p–p38	Guo and Ding (2018)
2	Cardiac glycoside HTF-1	Cervical cancer, breast cancer and liver cancer	HeLa, MCF-7 and HepG2	↑p-JNK and↓p-ERK1/2	Ma et al. (2018a, b)
3	Asparanin A	Endometrial carcinoma	EC	↓Ras, ↓Rap1, ↓p-MEK and ↓p-ERK	Zhang et al. (2021a, b, c)
4	Timosaponin AIII	Breast cancer	MDA-MB-2 and MCF231	↑p38	Zhang et al. (2020)
5	Dioscin	Colon cancer	HT-29	↑p-JNK and↑p–p38	Li et al. (2018a, b)

### Quinones

**Fig. 8:**
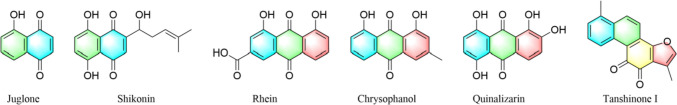
Structures of 6 quinones

Quinones are natural organic compounds that contain unsaturated cyclic diketone structures and mainly include four types: benzoquinone, naphthoquinone, phenanthrenequinone, and anthraquinone. Wang et al. (2018a, b, c, d) found that juglone could induce the activation of the p38 and JNK MAPK-signaling pathways, which partly led to autophagy in HepG2 cells, G2/M cell cycle arrest, and increased apoptosis in hepatocellular carcinoma cells. In addition, Han et al. (2019) observed that shikonin not only enhanced the phosphorylation of ERK, JNK, and p38 in a time-dependent manner, but also co-induced apoptosis in SNU-407 colon cancer cells through ER stress response and mitochondrial pathways. The anti-cancer mechanisms of action of quinones are shown in Table 7 and the structures are illustrated in Fig. 8.

**Table 7:** Detailed information of 6 quinones

No	Ingredients	Diseases	Use of cell lines	Mechanism of action	References
1	Juglone	Liver cancer	HepG2	↑p-JNK and↑p–p38	Wang et al. (2018a, b, c, d)
2	Shikonin	Colon cancer	SNU-407	↑p-JNK, ↑p–p38 and↑p-ERK	Han et al. (2019)
3	Rhein	Renal cell carcinoma	A489,786-O and ACHN	↑p-ERK and↑p-JNK	Ma et al. (2018a, b)
4	Chrysophanol	Oophoroma	ES2 and OVCAR3	↑p-ERK1/2 and↑p-JNK	Lim et al. (2018a, b)
5	Quinalizarin	Lung cancer	A549	↓p-ERK,↑p-JNK and↑p–p38	Meng et al. (2018)
6	Tanshinone I	Colorectal cancer	HCT116 and HT29	↑p–p38	Kim et al. (2018)

## Discussion

Recently, research on the supercritical extract of rosemary was reported and registered in a clinical trial (NCT05080920). Work in non-small cell lung cancer showed that it is used in the clinic alongside standard cancer drugs, such as cisplatin and parrolizumab. Supercritical extract of rosemary can inhibit the MAPK-signaling pathway and enhance immune anti-cancer functions. Therefore, further research into its use as an adjuvant in the treatment of non-small cell lung cancer is warranted (Bouzas et al. 2022).

Kittiwattanokhun et al. (2021) revealed that S. alata extract reduces the expression levels of MMP-3 and MMP-1353 in cells and inhibits chondrosarcoma SW1353 cell migration. These observed effects are also connected with inhibition of the MAPK-signaling pathway.

Chen et al. (2018a, b) evaluated the effects of the combined use of antrodia cinnamomea and ginger on liver cancer cells, specifically the HepG2 and Huh-7 cell lines. The results showed that antrodia cinnamomea and ginger synergistically inhibited the MAPK-signaling pathway, significantly reducing ERK and p38 phosphorylation. Their effects when combined were better than those of using antrodia cinnamomea alone. This is possibly because the coculture of antrodia cinnamomea and ginger produced new triterpenoids, which changed the active components of the original plant. This concept may provide a valuable new direction for cancer treatment development.

Various internal and external factors contribute to cancer evolution. In different types of cancer, genetic changes, dysregulated signaling pathways, and loss of internal loop homeostasis all collectively support tumor growth. Alterations to components of the MAPK-signaling pathway are often present in various cancers. It is a crucial pathway for cancer cell drug resistance and proliferation, and is becoming a potential therapeutic target.

Various protein kinases in the MAPK-signaling pathway have been used as predictive biomarkers in preclinical studies of various cancers. Many researchers have also attempted to identify effective drugs to target this pathway, some of which are in clinical trials. However, drug resistance and side effects are major problems associated with these drugs. Because of the safety of natural products, the roles of their various chemical ingredients are gradually being explored. These include chrysophanol, rhein, brasinin, fargesin, and apocynin, which have been found to potentially target the MAPK pathway and are in the development stage of preclinical trials.

However, although many single-component anti-cancer mechanisms have been reported, there are few studies on the combined treatment of the chemical components of various natural products or their use in combination with clinical anticancer drugs. According to the existing in vitro experiments, the anti-cancer effects of using a combination therapy are significantly higher than those associated with a single drug. Second, because of the limitations of clinical research, there are few natural products in the clinical trial stage. However, some of these natural products are better than the existing synthetic MAPK inhibitors in terms of safety and effectiveness. Better treatment strategies may be obtained using them as an adjuvant or routine treatment for cancer. Exploring derivatives of these natural ingredients may also be a potential way to enhance the anti-cancer capacity of the original compounds.

## Conclusion

The abnormal activation of specific proteins in the MAPK-signaling pathway is an important cause of various cancers. Therefore, therapeutically intervening in this signaling pathway may be an effective tumor treatment strategy. Considering the side effects of the existing MAPK inhibitors, exploring natural product compounds provides a new direction for cancer treatment. For example, vanillin, restoratrol, curcumin, oridonim, astragaloside IV, and ursolic acid have been extensively studied and found to have good anti-cancer effects. In the future, we should focus on the development of natural medicines, as well as their use in conjunction with existing medical technologies, to enhance cancer treatment approaches.

## Data Availability

Not applicable.

## Abbreviations

MAPKKK: Mitogen-activated protein kinase kinase kinase
MAPKK: Mitogen-activated protein kinase kinase
MAPK: Mitogen-activated protein kinase
ERK1/2: Extracellular regulated kinase 1/2
JNK: C-Jun N-terminal kinase
p38: P38 mitogen-activated protein kinase
ERK5: Extracellular regulated kinase 5
UCP1: Uncoupling protein 1
ROS: Reactive oxygen species
ECM: Extracellular matrix
TMEM2: Transmembrane protein 2
HA: Hyaluronic
ER: Endoplasmic reticulum
CD44: Cluster of differentiation-44
AICD: Activation-induced cell death
HSC: Hemopoietic stem cell
ISCs: Intestinal stem cells
EGFR: Epidermal growth factor receptor
PDAC: Pancreatic ductal adenocarcinoma
TRPM2: Transient receptor potential melastatin 2
HDACs: Histone deacetylases
